# Biomechanics of Lower Limbs during Walking among Candidates for Total Knee Arthroplasty with and without Low Back Pain

**DOI:** 10.1155/2015/142562

**Published:** 2015-06-11

**Authors:** David R. Burnett, Naira H. Campbell-Kyureghyan, Robert V. Topp, Peter M. Quesada

**Affiliations:** ^1^Speed School of Engineering, University of Louisville, Louisville, KY 40292, USA; ^2^College of Engineering and Applied Sciences, University of Wisconsin-Milwaukee, Milwaukee, WI 53211, USA; ^3^College of Nursing, Marquette University, Milwaukee, WI 53233, USA; ^4^Department of Mechanical Engineering, University of Louisville, Louisville, KY 40292, USA

## Abstract

The effect of joint pathologies, such as unilateral knee osteoarthritis (UKOA) or low back pain (LBP), on bilateral gait symmetry has gained increased attention during the past decade. This study is the first to compare gait patterns between patients with UKOA and LBP in combination and with UKOA only. Temporal, kinematic, and kinetic variables were measured bilaterally during gait stance phase in 31 subjects with UKOA and LBP (Group I) and 11 subjects with only UKOA (Group II). Group I patients exhibited less hip rotation in the affected limb (A) than in the nonaffected (NA) limb during walking in contrast to Group II patients. Group I patients had minimal bilateral differences in hip abduction and flexion, but Group II patients displayed significantly larger values in the NA limb compared to the A limb for both parameters. Hip flexion patterns were significantly different between Groups I and II. Subjects in both groups adapted gait patterns that minimized vertical ground reaction force, knee flexion motion, and stance time on the UKOA affected limb. The distinct kinematic gait patterns that were revealed in this study may provide clinical value for assessment of patients with UKOA in conjunction with LBP.

## 1. Introduction

Joint pain is experienced by most people at some point in their lifetime and seven out of eight sufferers report pain in multiple joints [1]. However, the focus of current research and clinical practice is typically directed at individual joints and does not consider the temporal or potentially contributing relationships between multiple joint pain within individuals [1]. Two areas of the body that are most commonly characterized by pain are the knee(s) and the spine.

Osteoarthritis (OA) can affect any joint in the body, but the medial tibiofemoral compartment is most commonly affected, and it was estimated to impact approximately 21 million Americans [2]. Low back pain (LBP) is another very prevalent problem, affecting over 30 million Americans [3–5], with the lifetime prevalence estimated at 75–85% [6]. Wolfe et al. [7] found that general back pain was present in 54.6% of knee osteoarthritis (KOA) patients while Anderson et al. [8] reported 21.8% comorbidity of KOA and LBP among 398 subjects. However, a limited amount of research exists regarding the potential temporal and biomechanical relationships between KOA and LBP [9]. Toriyama et al. [10] insisted that assessments of UKOA patients should consider not only the affected knee but also additional joints such as the opposite knee and hip. Additionally, McGregor and Hukins [11] suggested that the spine should not be viewed in isolation from the lower limbs, especially the hip and knee, in patients with LBP. Spatial and temporal parameters and kinematic patterns of gait have clinical value for the assessment of lower limb joint pathologies. The notion of bilateral symmetry/asymmetry during gait in asymptomatic (healthy) subjects, as well as patients with KOA, has been the focus of many researchers. Sadeghi et al. [12] suggest that symmetry is achieved if no statistical differences exist between parameters that are measured bilaterally. Another recent study by Collins et al. [13] found that muscle cocontraction and dynamic knee joint stiffness symmetry is maintained in subjects with early stage OA.

Kinematic and kinetic variables such as multiplanar lower extremity joint motion and ground reaction force (GRF) [14–19] have been analyzed in addition to comparisons of bilateral muscle activity using electromyography (EMG) [19–23]. While these studies often suggested that bilateral symmetry is common in asymptomatic individuals, conclusions do vary and a consensus has not yet been reached on the subject.

The effect of joint pathologies such as KOA or LBP on bilateral gait symmetry has gained increased attention during the past 10–15 years. Several previous studies have concluded that patients with KOA exhibited significant differences between the affected and nonaffected limbs during gait or stair ascending based on variables such as stance time and gait velocity [24–28], lower limb kinematics (knee range of motion, peak flexion/extension) [26, 28–30], and kinetics (GRFs, moments) [26, 29, 31]. Conversely, other researchers have indicated that significant differences in kinematic or kinetic parameters measured bilaterally did not exist among KOA patients [31–34]. While the research protocols utilized in these studies varied slightly as did the severity and progression of KOA, discrepancies in conclusions highlight the need for further research concerning bilateral symmetry in patients with KOA.

For studies of patients with LBP in isolation, varied results related to bilateral symmetry have also been found. Khodadadeh and Eisenstein [35] and Al-Obaidi et al. [36] found no bilateral differences in spatial-temporal measures such as stance time and step length during gait in patients with LBP while Lamoth et al. [37] and Simmonds et al. [38] concluded that differences in these measures did exist between the right and left limbs in LBP patients. Several research studies reported significant differences in GRFs between the right and left limbs in LBP patients [38–40]. A pair of studies by Lamoth et al. [41, 42] investigated EMG activity of back muscles measured bilaterally during gait in LBP patients and found no significant differences. Research focused on evaluating bilateral symmetry/asymmetry during gait in patients with LBP to date was viewed in isolation from the lower limbs and has not specifically quantified bilateral kinematic and kinetic differences.

Reduced range of motion in the lower limb joint during walking can be compensated by increased motion in the pelvis and torso. Consequently it may affect the natural motion of the lower back and initiate pain in the lumbar region of the spine because of their kinematic interaction. A thorough review of the literature has discovered that no study has attempted to compare bilateral biomechanical symmetry measures during gait between patients for TKA with UKOA only and UKOA and LBP in combination.

The purpose of this study was twofold: (1) to determine if differences in biomechanical symmetry exist between patients with unilateral knee osteoarthritis (UKOA) with and without LBP and (2) compare the gait alteration present in patients with unilateral UKOA and LBP. It was hypothesized that differences in biomechanical measures related to gait between limbs will be found in patients with UKOA both with and without LBP. It was further hypothesized that UKOA patients without LBP will exhibit the same biomechanical symmetry patterns in the knee joint as patients with UKOA and LBP during walking. This is based on the understanding that the knee joint is highly responsible for support and balance throughout the gait cycle [12, 43, 44]. Thus, the presence of UKOA will be the primary factor related to biomechanical symmetry of the knee regardless of whether or not the person has LBP. It was also hypothesized that patients that have UKOA in isolation will exhibit greater biomechanical symmetry in the hip joint in comparison to those with UKOA and LBP. The rationale for this hypothesis is linked to the presumed synergistic effects of multiple painful joints and the substantial role of the hip in propulsion that was established in previous literature [45–47]. Persons suffering from both UKOA and LBP may tend to develop more asymmetrical gait patterns than those with only UKOA in order to compensate for the dual nature of their pain.

## 2. Methods

### 2.1. Subjects

A total of 42 candidates for unilateral TKA related to OA were recruited for this study from a local orthopaedic clinic. All subjects were screened and referred by orthopaedic surgeons and voluntarily agreed to participate in the study by signing the consent form approved by the Institution Review Board (Protocols: #215.03 and #10.275).

Subjects were excluded if they reported a history of uncontrolled angina, cardiomyopathy severe enough to compromise cardiac functioning, any clinical history of lesion or surgery affecting a lower limb, hip or the lumbar spine, OA affecting any other joint of a lower limb (hip or ankle) or both knees, or any other neurological or health problem that inhibit moderate walking ability. The candidates for TKA were then separated into two groups based on their responses to a customized questionnaire concerning the existence of LBP. Group I consisted of 31 TKA candidates (8 males, 23 females) who were previously diagnosed with chronic LBP that lasted for 90 days or more. Group II consisted of eleven TKA candidates (7 males, 4 females) who did not report LBP. Burnett et al. [9] provide more specific methodological details concerning the pain related questions and criterion by which Group I and Group II were determined. All subjects were right leg dominant.  Table 1 provides a summary with respect to each group of subjects. Group differences were not apparent for demographic information (*p* > 0.05).

### 2.2. Biomechanical Data Collection and Processing

**Figure 1:**
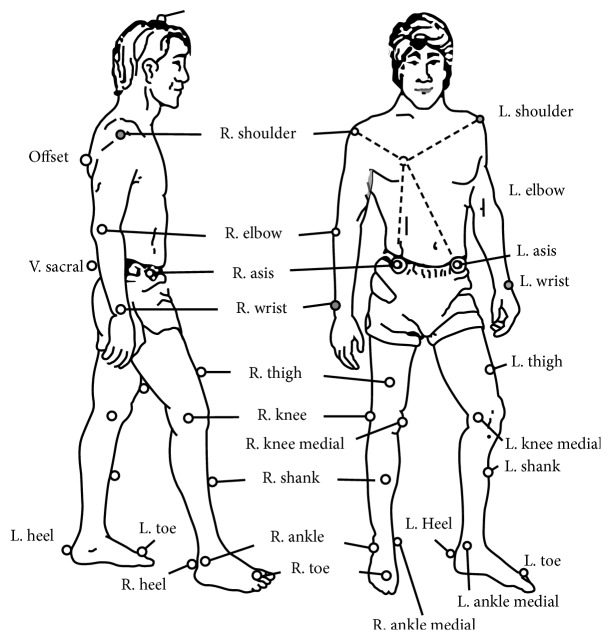
Modified Helen Hayes marker arrangement utilized for 3D motion analysis.

Data acquisition during walking trials for all subjects was performed at the biomechanics laboratory at the University of Louisville. Twenty-four reflective markers were placed on anatomical landmarks of the subject in a modified Helen Haynes marker arrangement (Figure 1; [48]). Three-dimensional motion capture was performed using an eight-camera motion tracking system (Motion Analysis Corp., Santa Rosa, CA) at a frequency of 100 Hz. Vertical ground reaction force (GRF) data was obtained while subjects walked across a 6-component force platform (Bertec Corp, Worthington) at 1000 Hz. EvART 7.0 and Cortex 2.2.1 were used to track marker trajectories and GRF during walking trials in which subjects walked at a self-selected pace and contacted the force plate as part of a 6-minute walk protocol [49].

The subject was instructed to direct his/her attention straight ahead and not to target the force plate, and the starting position was adjusted until he/she struck the force plate during a normal stride. During the 6-minute walk protocol, biomechanical data was captured for 3 instances with the subject's affected (A) leg contacting the force plate, and 3 instances with the nonaffected (NA) leg contacting the force plate. Lower limb dominance was determined by the foot used to kick a ball with maximum force. The right side was found to be dominant for all patients in Group I and Group II. The dominant leg was the affected leg in 61% and 73% of subjects Groups I and II, respectively.

Trials were randomized, and all subjects were given adequate rest time in order to minimize the effect of fatigue. 3D kinematic and kinetic data were processed using OrthoTrack 6.2.8. Temporal, kinematic, and kinetic variables were measured bilaterally during the stance phase of gait and were used to make comparisons between groups. Specific variables included peak vertical GRF (VGRF), stance time, and hip and knee range of motion (rotation, abduction, and flexion). Range of motion (ROM) was calculated by determining the difference between the largest and smallest angular values for rotation, abduction, and flexion.

### 2.3. Measures of Symmetry

Determination of symmetry was calculated using the symmetry index (SI) given by

(1) $$\begin{array}{c} SI=\frac{X_{A}}{X_{NA}}, \end{array}$$

where *X*_A_ is the gait variable recorded for the limb affected by pain and *X*_NA_ is the corresponding variable for the nonaffected limb. For each SI, a value of 1 would indicate perfect symmetry while values that deviate from unity indicate that one limb is being favored over the other. In order to make direct comparisons of symmetry between the groups, an additional calculation was performed to determine the absolute percent (%) difference of the SI from 1 (|1 − SI|).

Statistical differences in biomechanical parameters between groups were determined with one-way Analysis of Variance (ANOVA) with two treatments (Group I-II). An alpha level of 0.05 was used to determine significance. Paired *t*-tests at an alpha level of 0.05 were used to determine if the values measured for the NA limb were statistically different from the A limb. Minitab 16 Statistical Software (State College, PA) was used for statistical analysis.

## 3. Results

### 3.1. Between Limb Comparisons

Subjects in Group I and Group II exhibited significant (*p* < 0.05) differences in several parameters measured bilaterally in the NA and A limbs (Table 2). VGRF and knee flexion range of motion (ROM) were significantly greater in the NA limb compared to the A limb in both Group I and Group II. Hip rotation ROM (SI = 0.858; *p* = 0.001) and stance time (SI = 0.975, *p* = 0.034) were also significantly greater in the NA limb compared to the A limb in Group I subjects. Group II subjects also had significantly greater hip abduction ROM (SI = 0.916, *p* = 0.026) and hip flexion ROM (SI = 0.856, *p* = 0.001) in the NA limb compared to the A limb.

### 3.2. Between Group Comparisons

**Figure 2:**
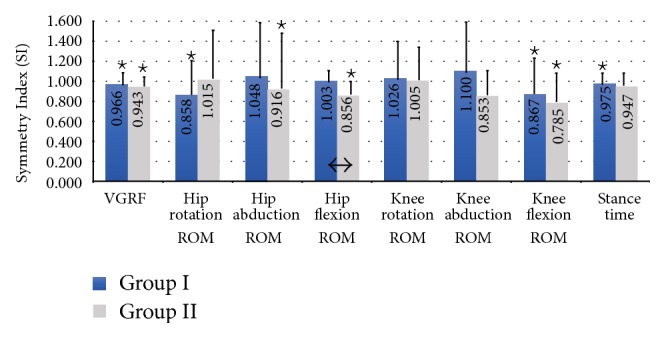
Comparison of vertical ground reaction force (VGRF), hip range of motion (ROM), knee ROM, and stance time symmetry between limbs and groups during gait stance phase. Significant differences (*p* < 0.05) in the biomechanical parameter measured in the surgically affected (a) limb compared to the nonsurgically affected (NA) limb indicated by an (*⋆*) displayed above the bar for that variable and that group. The significant differences between groups as determined by post hoc analyses are indicated by an arrow (↔).

There was a significant difference (*p* = 0.024) in hip flexion ROM symmetry between Group I (SI = 1.003) and Group II (SI = 0.856). Several other variables also exhibited contrasting symmetry patterns between Group I and Group II, although statistical significance was not achieved. Hip rotation ROM was greater in the NA limb compared to the A limb among Group I subjects (SI = 0.858) while Group II subjects (SI = 1.015) had smaller hip rotation ROM in the NA limb compared to the A limb. Opposing results (Group I SI > 1, Group II SI < 1) were also found between groups for hip abduction ROM, hip flexion ROM, and knee abduction ROM. Results are further described in Table 2 and Figure 2 with SI values shown as means.

## 4. Discussion

This study has uncovered a number of noteworthy findings with respect to eight bilateral biomechanical variables during the stance phase of gait in TKA candidates with and without LBP. Both groups of patients with UKOA exerted significantly less force on their A limb compared to their NA limb during the stance phase of gait (SI_GI_ = 0.966 and SI_GII_ = 0.943). Additionally, Group I subjects spent significantly less time on the A limb compared to the NA limb (SI = 0.975; *p* = 0.034). These finding are not surprising since all of these patients were scheduled for TKA within 4 weeks of the data collection session and would not want to exacerbate their pain by placing excessive force on that limb. This type of pain reduction mechanism was shown when these patients compensated by placing greater force and spending more time on their NA limb while completing the necessary task of supporting the body throughout the stance phase of walking. These VGRF compensation patterns for Group II patients are in accordance with several previous studies [26, 31, 33] while no study has considered this measure in patients with UKOA and LBP (Group I). It was also found that stance time in Group I and Group II patients in the current study as well as previous studies [25, 29, 50] was less for the A limb than the NA limb. This reduction in stance time between limbs provides further evidence to the notion that UKOA patients depend more on their NA limb during walking.

A specific aim of this study was to determine if subjects with UKOA and LBP in combination would exhibit distinct biomechanical differences during walking than patients that had UKOA in isolation. Examination of knee ROM in all three planes during gait stance phase revealed a number of interesting discussion points. The hypothesis that patients with UKOA will limit knee flexion ROM in their A limb in an effort to minimize pain in that limb is further supported by an evaluation and discussion of bilateral knee flexion ROM values for healthy subjects previously reported [14, 15, 19, 21, 51].

Based on the study by Burnett et al. [19] healthy subjects exhibited approximately 4% and 3% differences in knee flexion ROM, respectively, between the non-dominant (ND) and dominant (D) limbs during walking. Hence, in the absence of unilateral knee pain, they are utilizing essentially symmetrical knee flexion motion to propel themselves forward.

It was interesting to discover that transverse plane knee motion (i.e., rotation) in both Group I and Group II patients was essentially perfectly symmetrical (knee rotation ROM SI = 1.026 and 1.005, resp.). Knee rotation will presumably intensify pain in patients suffering from UKOA who will therefore try to limit this rotational activity in both legs during walking. Additionally, it would seem that rotation about the knee is ancillary motion that does not contribute to propelling the person forward while completing successive steps. These patients will therefore attempt to keep their knee fairly rigid in the transverse plane and mimic the action of their nonpainful knee. In a previous study it was found that significant differences exist in bilateral knee rotation ROM [19] even for healthy subjects, and the gait asymmetries in healthy subjects may be due to one lower limb being more responsible for stabilization or support [43, 44, 52, 53], balance [54], or body weight transfer [43] while the opposite limb contributes more to propelling the body forward during walking [12, 43, 44]. Hannah et al. [15] also found that transverse plane knee motion was the least symmetrical joint motion in healthy subjects during walking.

When bilateral knee flexion angles are considered, however, it is apparent that both Group I and Group II subjects reduced the amount of motion in their A limb compared to their NA limb. Similar results for bilateral knee flexion ROM in patients with only UKOA have been previously reported [26, 30, 55, 56], while the current study is the first to report such findings in patients with UKOA and LBP (Group I). Knee flexion motion will be a primary contributor to propulsion during walking, and it would seem that this propelling activity will be difficult and painful for UKOA patients to perform, particularly with their A leg [28, 55, 57]. In order to continue progressing forward during walking, UKOA patients will depend more on their NA limb to perform this propulsive movement which will increase the knee flexion motion for the NA limb.

Group I patients exhibited 14.2% (*p* = 0.001) less hip rotation in their A limb than their NA limb during walking while this parameter was 1.5% larger in the A limb compared to the NA limb in Group II patients. However, Group I patients had minimal bilateral differences in hip abduction and hip flexion, but Group II patients displayed significant differences between the A and NA limbs for both of these biomechanical variables (SI_GII_ = 0.916, *p* = 0.026 and SI_GII_ = 0.856, *p* = 0.001, resp.).

When persons have pain in their lower back they will maintain essentially symmetrical hip motion in the frontal and sagittal plane while rotating one hip more than the other [45, 47, 58]. These mechanisms may be the result of an altered gait pattern they have adapted over time to reduce or minimize their pain during walking [45]. Adaptations in motion at the hips may contribute to additional modifications further down the kinematic chain (i.e., from the hips to the knee). This potential contributory relationship is further indicated by the fact that patients with a coexistence of UKOA and LBP continued to display discrepancies in hip rotation ROM between limbs. These results indicate that future studies of relationships between LBP and UKOA should involve assessment of multiple lower extremity joint kinematics to determine if asymmetries exist.

Group II subjects exhibited significantly reduced hip abduction and hip flexion ROM in their A limb compared to their NA limb. By acquiring this type of asymmetrical hip motion in the frontal and sagittal plane, patients in Group II are presumably aiming for a reduction in pain when their A limb is fully supporting their entire body weight during walking [57]. This asymmetry in hip abduction and hip flexion ROM in Group II patients differs from Group I patients who will have to consider the presence of pain in their back in addition to their knee. Group II patients therefore will not produce the same biomechanical tendencies and can exhibit larger movement about their NA hip while walking since these motions will not be affected by the existence of LBP.

These types of biomechanical differences between UKOA patients with or without LBP have never been previously reported, and studies that considered bilateral hip motion in multiple planes are rare. Thus it is not possible to directly compare this particular finding with other studies that used a similar population of subjects. Nevertheless, in a study by Briem and Snyder-Mackler [55], significantly smaller hip flexion angles were found for the uninvolved limb during weight acceptance in 32 patients with moderate knee OA. Their findings also differ from the current study with respect to hip abduction. Briem and Snyder-Mackler [55] reported that hip abduction was greater for the involved limb compared to the uninvolved limb when the heel first strikes the floor and remained relatively more abducted throughout stance phase. Discrepancies in results between the current study and the study by Briem and Snyder-Mackler [55] may potentially be due to difference in UKOA severity among subject groups. Subjects in the current study were all scheduled for TKA surgery within 4 weeks of the data collection session and could have been more likely to favor their NA limb compared to subjects in the previous study which were reported to have “moderate” UKOA and being fitted for a brace.

This study has several limitations. First, the study did not include muscle activity analysis. Future study is needed to analyze the effect of multiple joint pain on muscle response. Second, the asymmetry patterns may differ in magnitude with varying degrees of knee OA in coexistence with LBP. In the future it will be beneficial to investigate this with more extensive study design. Third, the cross-sectional design of this study created an imbalance in sample number between groups.

## 5. Conclusion

The study is the first to investigate biomechanical symmetry measures bilaterally during walking in candidates for TKA with and without LBP. This research has investigated and confirmed the hypotheses that patients with UKOA and LBP will exhibit distinct patterns of biomechanical symmetry/asymmetry differing from patients with only UKOA. This finding may shed new light on differences between various gait parameters of UKOA with and without LBP. It was revealed that patients with UKOA and LBP exhibit significantly different gait strategies. Most gait alterations in UKOA patients with LBP were characterized by reduced hip rotation, increased hip abduction, and increased knee abduction. The distinct kinematic patterns of gait that were revealed in this study may present a clinical value for the assessment of patients with TKA in conjunction with LBP.

## Figures and Tables

**Table 1:** Group demographic information.

Group	LBP	UKOA	Sample size	Age (avg. ± SD) years	Height (avg. ± SD) cm	Weight (avg. ± SD) kg
I	Yes	Yes	31	63.1 ± 7.26	167.1 ± 10.7	97.9 ± 29.2
II	No	Yes	11	63.0 ± 10.0	170.8 ± 11.3	99.9 ± 16.4

**Table 2:** Results of bilateral gait analysis parameters in Group I and Group II subjects.

Variable	Group	Symmetry index	Between limb *p* value	Between group *p* value
VGRF	Group I	0.966	***0.023***	0.714
	Group II	0.943	***0.012***	

Hip rotation ROM	Group I	0.858	***0.001***	0.368
	Group II	1.015	0.214	

Hip abduction ROM	Group I	1.048	0.273	0.726
	Group II	0.916	***0.026***	

Hip flexion ROM	Group I	1.003	0.494	***0.024***
	Group II	0.856	***0.001***	

Knee rotation ROM	Group I	1.026	0.849	0.317
	Group II	1.005	0.795	

Knee abduction ROM	Group I	1.100	0.640	0.251
	Group II	0.853	0.109	

Knee flexion ROM	Group I	0.867	***0.001***	0.553
	Group II	0.785	***0.050***	

Stance time	Group I	0.975	***0.034***	0.170
	Group II	0.947	0.118	
